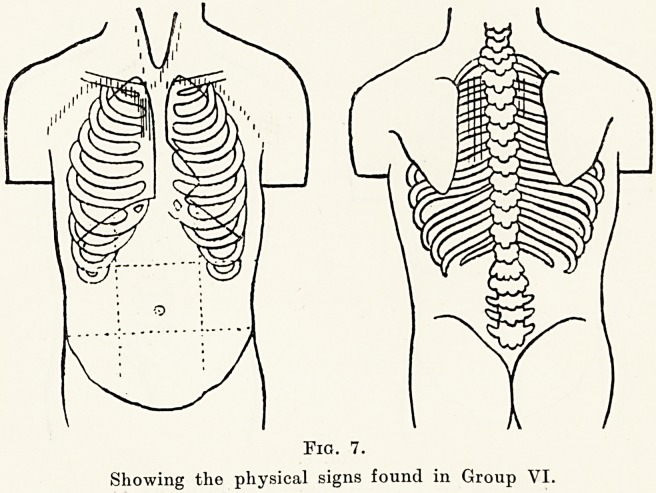# Congenital Heart Disease as Seen in Elementary School Children
*Read to the Bristol Medico-Chirurgical Society on 10th December, 1930.


**Published:** 1931

**Authors:** C. Bruce Perry

**Affiliations:** Medical Registrar, Bristol General Hospital; Colston Research Scholar; Assistant Physician, Children's and Winford Hospitals. (From the University Centre of Cardiac Research, Bristol General Hospital.)


### CONGENITAL HEART DISEASE AS SEEN IN ELEMENTARY SCHOOL CHILDREN.*

BY

C. Bruce Perry, M.D., M.R.C.P.,

*Medical Registrar, Bristol General Hospital;*
*Colston Research Scholar;*
*Assistant Physician, Children's and Winford Hospitals.*

(*From the University Centre of Cardiac Research, Bristol General Hospital.*)

---

This paper is based on a review of 121 children referred by the School Medical Service for an opinion on their cardiac condition, and in whom the signs found were considered to be due to a congenital malformation of the heart. They have been divided into groups according to their physical signs, which will be dealt with more fully in considering the individual groups. An idea of the incidence of these conditions may be gathered from the fact that there are in Bristol about 15,500 school children, which (assuming that none have escaped detection) gives an incidence of about 0·22 per cent. Actually it is probably a little higher than this.

Symptoms definitely attributable to the cardiac lesion, such as cyanosis, finger clubbing and dyspnœa, were observed in five, or 4·1 per cent. Attacks of cyanosis and dyspnœa on exertion were found in a further sixteen, or 13·3 per cent. Seven gave a history of "heart attacks." These attacks are very puzzling. As a rule the child is said to go pale suddenly,

* Read to the Bristol Medico-Chirurgical Society on 10th December, 1930.

to be dazed for a time, and to recover by an attack of crying. One child in this series was definitely epileptic. It was considered wise to advise restriction of games and drill at school in nine cases, or 7·5 per cent., and only one child was unable to attend school at all.

In view of the fact that in infants congenital heart disease often accounts for otherwise unexplained marasmus, it was thought worth while to compare the weights of these children with the average weights for their age. This comparison is shown in Figure 1, in which the curve for the average normal weights is taken from that given in Robert Hutchison's *Lectures on Diseases of Children.* There is obviously no very marked stunting in the growth of these children. The few low weights were evenly distributed amongst all the groups, and no one physical sign was found to be associated with a stunted child.

FIG. 1.

Showing ages and weights compared with the normal curve.

Only one child was found to be markedly backward, and that was a child who, although attending an ordinary school, at the age of 5½ had some difficulty in talking. The sex incidence shows a slight preponderance of males, there being 70 boys and 51 girls in the series. There were three cases of family incidence, when two members of the same family were found to have signs of a congenital cardiac malformation. In two of these the signs were similar in the two children, and in one they differed.

In one interesting family the eldest of three, a girl, had definite signs but no symptoms.

The next child, a boy, had more marked signs, moderately severe symptoms. A younger sister, who when examined had no signs, was shortly afterwards killed in a motor accident. Post-mortem examination revealed no cardiac deformity, but at the site of the junction of the obliterated ductus arteriosus the pulmonary artery showed a funnel-shaped depression about an eighth of an inch deep. She had apparently just escaped a patent ductus arteriosus.

*Group I.*—This is composed of 41 children, 25 boys and 16 girls. In these the one common physical sign was a systolic murmur varying in intensity, but always maximal at the inner end of the third or fourth left rib spaces. While this murmur may be audible all over the præcordium, it is never heard in the back. It is accentuated on auscultation of the patient in the "crawling" position. Some of these cases showed in addition a central systolic thrill, but in none was there any clinical cardiac enlargement. All of these children attended school normally and did the usual games and drill.

One was an epileptic, and two complained of occasional "heart attacks" such as I have already described. Three gave a not very definite history of dyspnœa on exertion, and two of vague præcordial

FIG. 2.
Showing the physical signs found in Group I.

FIG. 3.
Showing the physical signs found in Group II.

pain. One had a rather high colour, and is the child I have already mentioned, who was not talking normally at the age of $5\frac{1}{2}$.

*Group II.*—This group consists of 5 children, and differs from the first in that while the murmur is maximal in the same position it is also heard at and above the pulmonary area and in the left back.

Again, none of these showed any clinical evidence of cardiac enlargement. One of them had a high colour, clubbed fingers, and became cyanosed and dyspnœic on exertion, and while attending school was unable to do games and drill.

One other also had a high colour and suffered from attacks of dyspnœa and cyanosis, but was able to attend school normally. All five of the children in this group were boys.

*Group III.*—There were 41 children in this group, 23 boys and 18 girls. The sign characteristic of these is a systolic murmur maximal at and above the pulmonary area and audible in the back. Most of them also have an accentuated pulmonary second sound. Six showed clinical signs of cardiac enlargement in the shape of increased cardiac dullness. Four of these children were cyanosed, and of these two also had clubbing of the fingers.

Six became cyanosed or dyspnœic on exertion, and a further three were noticed to have a high colour. Four of the cases in this group complained of "heart attacks." It was considered advisable that one of these children should not attend school at all, and five others were advised to do no games or drill. All the others attended school normally with no ill-effects.

FIG. 4.
Showing the physical signs found in Group III.

FIG. 5.
Showing the physical signs found in Group IV.

*Group IV.*—This group was composed of 23 children, 11 boys and 12 girls. The sign characterizing this group is exactly similar to that of Group III, except that the murmur is not audible in the back.

Two of these showed in addition signs of slight cardiac enlargement, two were noted to have a high colour, and three were said to become dyspnœic on exertion. One complained of "heart attacks."

One was advised to play no games on account of the cardiac enlargement, but when last I saw him I was informed that he had won several races in the sports last summer. He was apparently none the worse for it.

*Group V.*—This comprised 8 children, 4 boys and 4 girls. The signs in this group were a general systolic murmur maximal at the pulmonary area, where it becomes continuous with a diastolic murmur, producing the so-called "machinery" or "humming-top"

FIG. 6.
Showing the physical signs found in Group V.

murmur. The systolic part of the murmur is audible in the back, and the second sound at the pulmonary area is very accentuated. There is also a general systolic thrill all over the præcordium. Five of the children in this group showed also signs of cardiac enlargement.

There was no cyanosis. All attended school, but three were exempted from games and drill.

*Group VI.*—This group is formed by 3 cases, 2 boys and 1 girl. The physical signs of this group consist of a soft general systolic murmur which becomes much more marked at the aortic area, and is conducted into the neck, into the back, and in one case at least can be heard along the brachial arteries.

The apex-beat, while normally situated, is rather shock-like on palpation, and the first sound at the apex is very accentuated. The blood-pressure is within normal limits. None of these cases had any symptoms of disability.

FIG. 7.

Showing the physical signs found in Group VI.

While it is difficult to assign any definite anatomical lesion to these various clinical signs, I venture to put forward the following suggestions. In the first group the systolic murmur maximal at the inner end of the third and fourth left spaces is, I think, probably due to a patency of the inter-ventricular septum. In Group II the murmur, which is similar, but heard also in the back, is probably due to the same condition more pronounced, but it has been separated in view of the uncertainty. The systolic murmur maximal at the pulmonary area characteristic of Group IV is probably due to a pulmonary stenosis, but whether the conduction of this murmur through to the back, as in Group III, is merely a more severe degree of this, or indicates the association of pulmonary stenosis with some other lesion, such as a septal defect, it is difficult to say. Post-mortem such a condition is often found, but the difficulty is that post-mortems are usually performed on patients showing far more severe symptoms. It is quite possible that in these two groups have been included several different types and combinations of anatomical defect. The signs of Group V are those characteristic of a patent ductus arteriosus with the "humming-top" murmur. The loud aortic systolic murmur conducted along the course of the larger arteries, heard in Group VI, probably indicates a sub-aortic stenosis.

These conditions have to be diagnosed from hæmic or functional murmurs and rheumatic heart disease. As regards the former, the murmur itself is different in quality, and is not so sharply localized as the murmur of a congenital malformation, and also it is not conducted.

The murmur of congenital defects is constant both in position and character. Many of these children have been watched over a space of three years, and only minor alterations have been noted, if any at all. In rheumatic heart disease the murmurs have a very different localization, and are usually associated with evidence of cardiac enlargement and an alteration in the character of the sounds themselves.

The criteria on which these children have been debarred from games and drill at school are definite cyanosis and dyspnœa, or anything more than a slight degree of cardiac enlargement.

Most of the children in this series have been investigated by X-rays and electrocardiography, but although my analysis of these records is not yet complete, I feel quite safe in stating that neither of these adjuvants is of much value in the differential diagnosis of congenital from other cardiac lesions.

This collection of cases has not been followed long enough to be valid from the point of view of prognosis. One knows that all of them are possible candidates for bacterial endocarditis, but what proportion of them will ultimately succumb to this or to some form of cardiac failure it is impossible to say. It is interesting to note that this small series of cases shows that only 7·5 per cent. have any serious disability, and most of these disability only to a very slight degree.

Since they have successfully reached school age, there would appear no reason why these children should deteriorate, unless perhaps through being given some excessively laborious occupation.

## Figures and Tables

**Fig. 1. f1:**
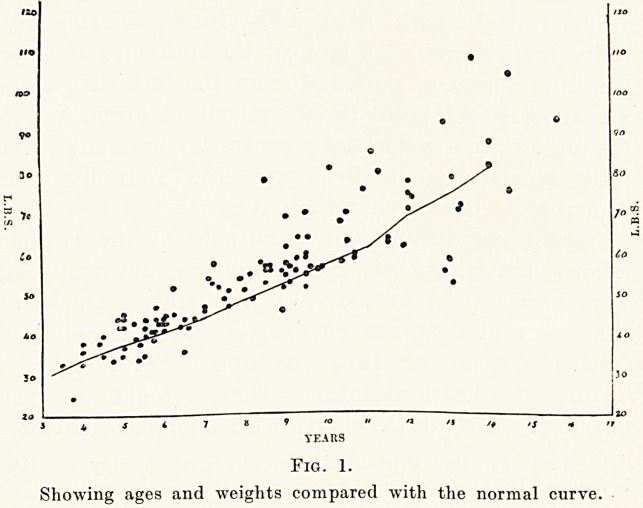


**Fig. 2. f2:**
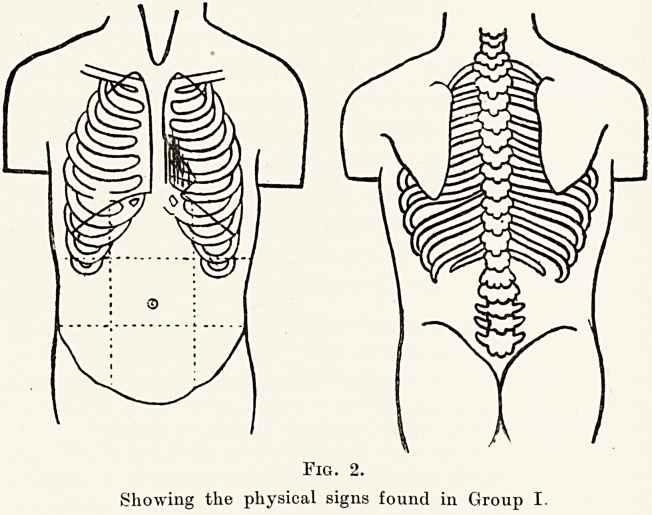


**Fig. 3. f3:**
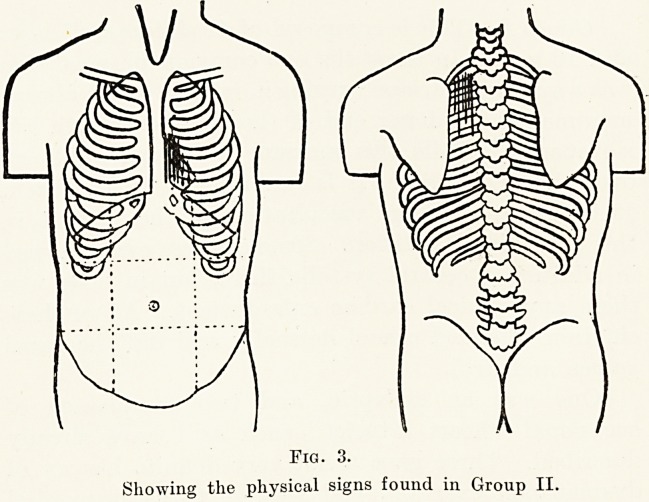


**Fig. 4. f4:**
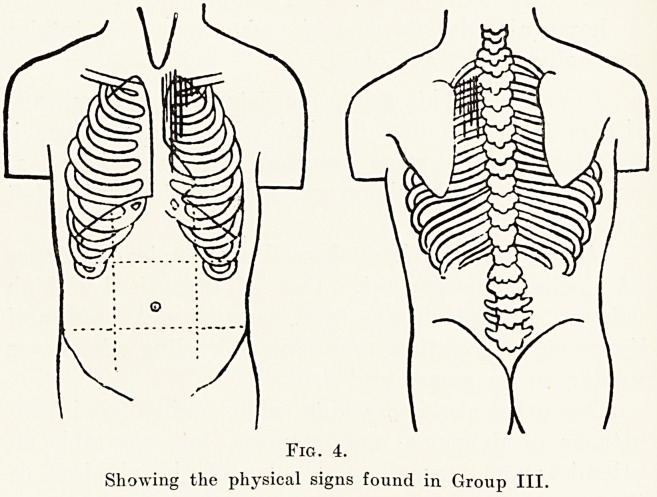


**Fig. 5. f5:**
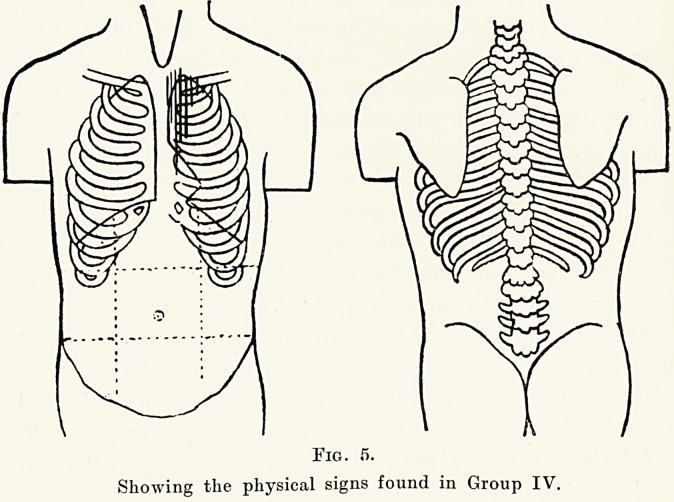


**Fig. 6. f6:**
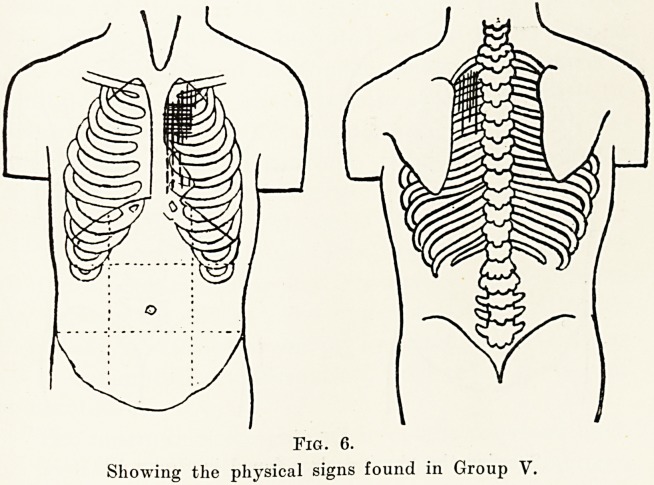


**Fig. 7. f7:**